# Case Report: Triple Primary Malignant Tumors of the Esophagus, Stomach, and Colon in a Patient With Genetic Analysis

**DOI:** 10.3389/fgene.2021.676497

**Published:** 2021-07-09

**Authors:** Xiaoli Zhan, Lingzhe He, Kai Song, Shuliang Cao, Erhong Meng, Yuedong Wang

**Affiliations:** ^1^Department of General Surgery, The Second Affiliated Hospital Zhejiang University School of Medicine, Hangzhou, China; ^2^Department of Pathology, The Second Affiliated Hospital Zhejiang University School of Medicine, Hangzhou, China; ^3^ChosenMed Technology (Beijing) Co. Ltd., Beijing, China

**Keywords:** multiple primary malignant tumors, esophagus cancer, gastric cancer, colon cancer, genetics

## Abstract

The incidence of multiple primary malignant tumors (MPMTs) has increased greatly with the progress of tumor diagnosis and therapy technology. However, triple primary cancer is still very rare, and its genetic change is not clear yet. This case report described a 70-year-old Chinese male patient with triple primary cancers of the esophagus, stomach and right-sided colon. Pathological examination confirmed that each malignant tumor developed independently. Next-generation sequencing (NGS) using a 599-gene panel revealed five *TP53* mutations in three tumor tissues. These variations might contribute to development of the triple primary malignant tumors in the patient. The patient underwent laparoscopic feeding jejunostomy and postoperative radiotherapy for synchronous esophageal and gastric carcinomas. Then, he underwent laparoscopic-assisted resection of right-sided colonic cancer and lysis of abdominal adhesions. By the time of submitting this manuscript, the patient had been well and no sign of recurrence or metastasis had been observed. To the best of our knowledge, this case is the first one to clarify the genetic abnormalities of triple primary cancers of esophagus, stomach and colon in a Chinese patient. It may contribute to understanding the molecular pathogenesis of multiple primary digestive malignancies and providing valuable treatment strategies for the similar patients in the future.

## Background

With the development of tumor diagnosis and treatment technology, the incidence of multiple primary malignant tumors (MPMTs) has increased greatly (Kumagai et al., 2001). However, triple primary cancer is still a very rare finding, and its genetic change is not clear yet. In this study, we reported the case of a 70-year-old Chinese man with triple primary cancers of esophagus, stomach and right-sided colon and clarified the genetic abnormalities underlying them.

## Case Presentation

**Figure 1:**
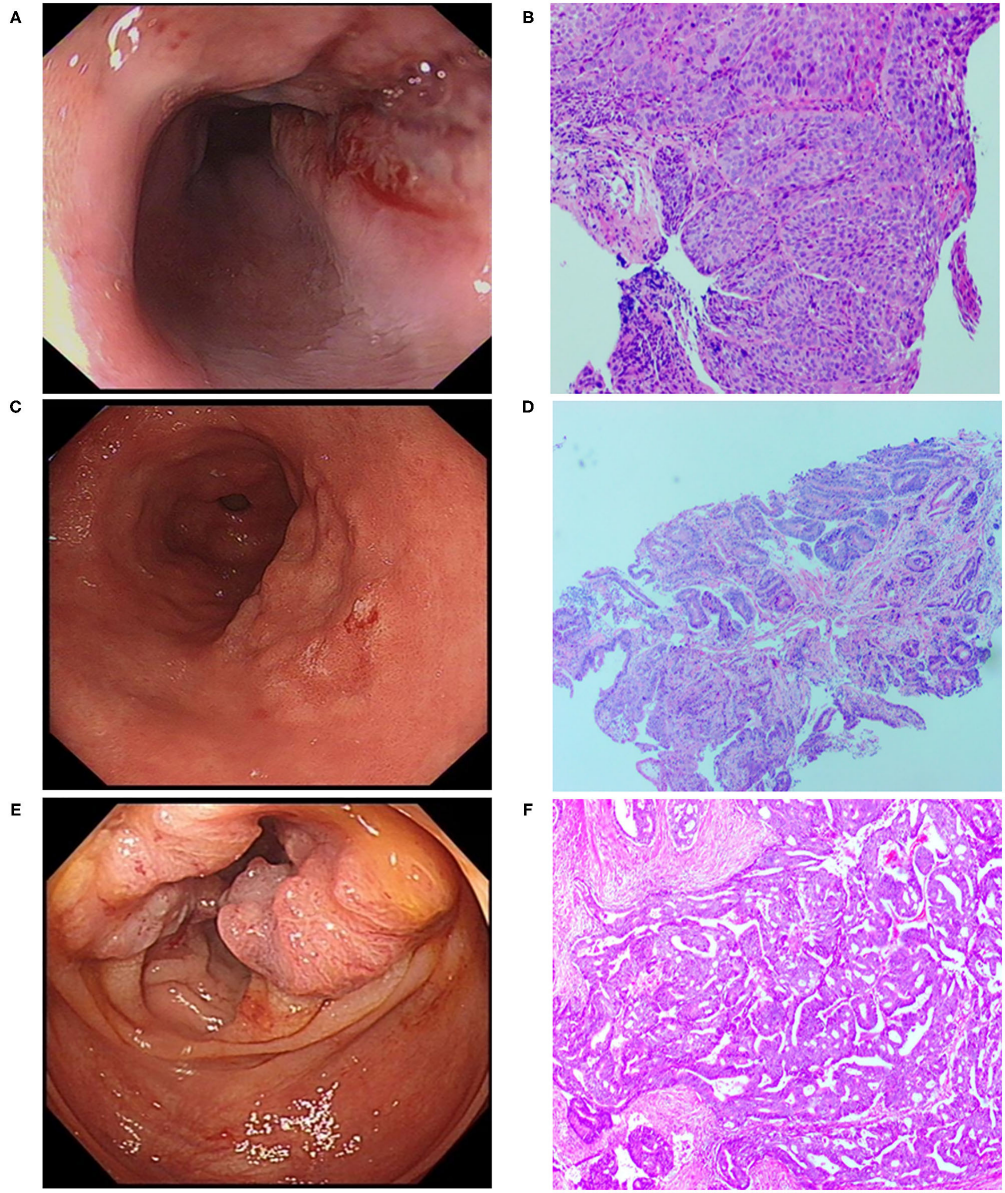
Esophagogastroduodenoscopic, Colonoscopic, and microscopic morphology of esophageal carcinoma, gastric carcinoma as well as colon cancer. **(A)** Esophagogastroduodenoscopic morphology of esophageal carcinoma. **(B)** Pathological examination showed esophageal squamous cell carcinoma (H.E. × 100). **(C)** Esophagogastroduodenoscopic morphology of gastric carcinoma. **(D)** Pathological examination revealed well-differentiated gastric adenocarcinoma (H.E. × 100). **(E)** Colonoscopic morphology of right-sided colon. **(F)** Pathological examination showed moderately differentiated adenocarcinoma of colon (H.E. × 100).

A 70-year-old male patient was admitted to our hospital complaining of choking while eating for more than 1 month on March 20, 2019. CT showed tumor signs in the middle of esophagus on March 6, 2019. Esophagogastroduodenoscopy demonstrated the patient was suffering from esophageal carcinoma and gastric cancer (Figures 1A,C). He had no family history of malignancy. Pathology examination confirmed they were locally advanced esophageal squamous cell carcinoma and adenocarcinoma of gastric horn (Figures 1B,D). On March 21, 2019, the patient underwent laparoscopic feeding jejunostomy and postoperative radiotherapy.

**Figure 2:**
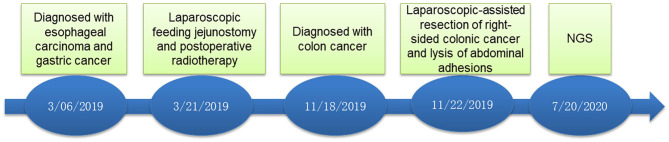
Timeline of the patient.

On November 15 and 18, 2019, esophagogastroduodenoscopy and abdominal CT showed that esophageal and gastric cancers were under control. However, CT indicated a tumor in the right-sided colon. On November 19, 2019, colonoscopy and pathological findings confirmed it was locally advanced moderately differentiated adenocarcinoma of colon (Figures 1E,F). Immunohistochemical stains revealed proficient mismatch repair proteins and positive expression of S-100, but negative expression of D2-40 or CD34. The patients underwent laparoscopic-assisted resection of right-sided colonic cancer and lysis of abdominal adhesions on November 22, 2019. By the time of submission of this manuscript, the patient had been well and no sign of recurrence or metastasis had been detected. The timeline was shown as Figure 2.

In order to seek possible personalized therapy strategies, the paraffin-embedded tissue blocks from malignant esophagus, stomach and right-sided colon, respectively, were subjected to next-generation sequencing (NGS) using a pan-cancer 599-gene panel [ChosenOne599, ChosenMed Technology (Beijing) Co. Ltd, Beijing, China] on July 20, 2020. NGS demonstrated the TMB value was 10.11 Muts/Mb, 11.37 Muts/Mb, 6.32 Muts/Mb in the tumor tissues of esophagus, stomach and colon, respectively, which ranked 29.58% (moderate), 6.45% (high) and 62.73% (low) in patients with corresponding malignancy. *TP53* mutation was identified in all three tumor tissues. However, the mutation location was different from each other. In the esophagus cancer tissues, two *TP53* variations were identified (NM_000546: exon6: c.580C>T: p.L194F and NM_000546: exon8: c.814G>A: p.V272M). In the gastric carcinoma tissues, there were also two *TP53* alterations (NM_000546: exon4: c.375G>A: p.T125T and NM_000546: exon7: c.715A>G: p.N239D). In the right-sided colon carcinoma tissues, there was one *TP53* mutation (NM_000546: exon8: c.821T>G: p.V274G) (Table 1).

**Table 1:** Genetic alterations detected in the patient's tumor tissues.

**Sample**	**Gene**	**Transcript**	**Exon**	**Nucleotide change**	**Alteration**	**Mutant allele frequency/Copy number**
Esophagus carcinoma	TP53	NM_000546	6	c.580C>T	p.L194F	19.50%
	TP53	NM_000546	8	c.814G>A	p.V272M	13.30%
	ATRX	NM_000489	21	c.5424T>G	p.Y1808*	4.65%
	CHEK1	NM_001114121	7	c.676dup	p.T226fs	3.23%
	EPHA7	NM_001288629	10	c.1787A>T	p.K596I	5.90%
	FBXW7	NM_033632	10	c.1463T>C	p.I488T	9.90%
	INHBA	NM_002192	3	c.992A>G	p.K331R	2.20%
	INPPL1	NM_001567	5	c.608A>C	p.H203P	3.20%
	NOTCH1	NM_017617	22	c.3640C>G	p.Q1214E	7.90%
	POLD1	NM_002691	15	c.1823C>T	p.P608L	3.40%
	PRKAR1A	NM_002734	9	c.883_891del	p.295_297del	20.50%
	RB1	NM_000321	21	c.2186A>G	p.K729R	2.53%
	TET1	NM_030625	4	c.2640A>T	p.L880F	6.90%
	CCND1				Gene amplification	12.5
	FGF19				Gene amplification	12.5
	FGF3				Gene amplification	12.5
	FGF4				Gene amplification	12.5
Gastric carcinoma	TP53	NM_000546	4	c.375G>A	p.T125T	11.00%
	TP53	NM_000546	7	c.715A>G	p.N239D	12.00%
	ATM	NM_000051	29	c.4315C>T	p.L1439F	6.00%
	ATR	NM_001184	18	c.3573G>T	p.L1191F	2.30%
	CARD11	NM_032415	8	c.1030_1032del	p.K344del	29.80%
	CARM1	NM_199141	10	c.1196+2T>G	Splicing mutation	22.60%
	CCND1	NM_053056	4	c.682C>T	p.R228C	2.20%
	CHEK1	NM_001114121	7	c.676dup	p.T226fs	3.43%
	GRM3	NM_000840	3	c.709C>T	p.R237C	33.10%
	PRDM14	NM_024504	2	c.215G>A	p.R72Q	11.60%
Right-sided colon carcinoma	FBXW7	NM_033632	9	c.1394G>A	p.R465H	22.10%
	PIK3CA	NM_006218	2	c.323G>A	p.R108H	20.40%
	PTEN	NM_000314	7	c.801+2T>G	Splicing mutation	2.18%
	TP53	NM_000546	8	c.821T>G	p.V274G	27.50%
	APC	NM_000038	16	c.2621C>G	p.S874*	23.00%
	APC	NM_000038	16	c.4468del	p.H1490fs	21.60%
	ARID5B	NM_032199	6	c.1004G>C	p.R335T	2.06%
	LATS2	NM_014572	5	c.2248G>C	p.G750R	8.00%
	NCOR1	NM_006311	19	c.2080C>T	p.R694*	30.50%
	PALB2	NM_024675	4	c.719C>T	p.P240L	8.10%
	TET1	NM_030625	4	c.4036T>G	p.L1346V	19.70%

At the same time, other important variations were also identified in the esophagus carcinoma tissues, including *ATRX* (NM_000489: exon21: c.5424T>G: p.Y1808^*^), *CHEK1* (NM_001114121: exon7: c.676dup: p.T226fs), copy number variations (CNVs) of *CCND1* and *FGF3/4/19*. In the gastric carcinoma tissues, the same *CHEK1* variation was also detected. In the colon carcinoma tissues, there were two *APC* variations (NM_000038: exon16: c.4468del: p.H1490fs and NM_000038: exon16: c.2621C>G: p.S874^*^, Table 1).

## Discussion

MPMTs are referred to the presence at least two histologically distinct malignancies that are not caused by recurrence or metastasis in the same patient. For the diagnosis of two primary malignant tumors, the following conditions must be met: (Dranka-Bojarowska and Lewinski, 2019) (a) Pathology confirms that both are malignancies; (b) Malignancies must locate separately. If they are close, they must be separated by a healthy mucosa with an area of at least 2 cm. If they are formed in the same organ, the period of more than 5 years after diagnosis must pass; (c) The possibility that the second malignancy is caused by metastasis from the primary site is excluded.

There are two kinds of MPMTs: synchronous and metachronous MPMTs. The former is defined as the diagnosis of a second primary malignancy within 6 months of the first primary malignancy, while the latter refers to the second primary malignancy diagnosed over 6 months after the first primary one (Zhai et al., 2018). Studies revealed that MPMTs often occur in the digestive system, followed by digestive-respiratory system in China (Zhai et al., 2018). The patient in our study was firstly diagnosed as esophagus carcinoma, then as gastric adenocarcinoma within one month (synchronously), finally as colon cancer 8 months later (metachronously).

To date, the molecular pathogenesis of MPMTs remains elusive. Some studies speculated that the development of MPMTs was associated with unhealthy lifestyle, genetic susceptibility, side effects of chemotherapy and radiotherapy, weak immunity, etc. The patient had a long history of smoking and drinking, and it might be one of predisposing factors of his MPMTs.

Studies demonstrated cancer develops from the accumulation of mutations in oncogenes and tumor suppressor genes (Califano et al., 1996). It is in line with this study, as the NGS data demonstrated the TMB value was middle to high, i.e., 10.11 Muts/Mb, 11.37 Muts/Mb, 6.32 Muts/Mb in the tumor sample of esophagus, stomach and colon, respectively, which ranked 29.58, 6.45, and 62.73% in the patients with corresponding malignancy.

Study of Dranka-Bojarowska and Lewinski (2019) revealed that genetic background, especially mutation of tumor suppressor gene *TP53, BRCA1*-associated protein 1 (*BAP1*) (Cheung et al., 2015) or partner and localizer of *BRCA2* (*PALB2*) (Schrader et al., 2016) played a crucial role in the development of MPMTs. Germline *TP53* mutation is closely associated with Li-Fraumeni Syndrome, characterized by a high frequency of various malignancies (Consul et al., 2020). In the patient, five different *TP53* mutations were detected in the patient's three tumors samples. Currently, clinical trial on Adavosertib (AZD1775, NCT02448329) targeted against *TP53* variations is ongoing.

In addition, *ATRX* variation (NM_000489: exon21: c.5424T>G: p.Y1808^*^) was also detected in the esophagus carcinoma tissues. According to the annotations of OncoKB, the *ATRX* mutation may be pathogenic. Previous studies have shown that loss of *ATRX* is associated with a variety of tumors such as sarcomas, hepatic angiosarcomas, gliomas and so on (de Wilde et al., 2012; Marinoni et al., 2014; Qadeer et al., 2014; Leeper et al., 2015; Liau et al., 2015a,b,c; Rodriguez et al., 2016; Singhi et al., 2017). There is currently no targeted therapy against the *ATRX* mutation.

The patient harbored *CHEK1* (NM_001114121: exon7: c.676dup:p.T226fs) in the tissues of esophagus carcinoma and gastric carcinoma (Table 1), Previous studies have shown that *CHEK1* mutation was associated with multiple malignancies, including lung cancer, prostate cancer, cervical cancer, and colorectal cancer (Gali-Muhtasib et al., 2008; Mazumder Indra et al., 2011; Al Nakouzi et al., 2014; Liu et al., 2015). According to the annotations of OncoKB, the *CHEK1* mutation may lead to loss of protein function, which is possibly pathogenic. However, no targeted therapy against the *CHEK1* mutation has been approved.

Besides, the patient also harbored CNVs of *CCND1* and *FGF3/4/19* (Table 1). *CCND1* often co-amplified with *FGF3/4/19*. A study reported that 86.7% (26/30) of the patients with esophageal carcinoma showed positive expression of CCND1 through immunohistochemistry (Hu et al., 2016). Clinical trials on Palbociclib (NCT04439201) and Abemaciclib (NCT03356223) targeted against *CCND1* amplification are ongoing.

In the colon carcinoma tissues, two *APC* alterations were detected (Table 1). According to the annotations of OncoKB, both *APC* mutations could cause protein loss of function, abnormal Wnt/β-catenin signaling pathway and promote the development of colon cancer. To date, No targeted drug against these two *APC* mutations have been approved.

Germline mutations were not mentioned in this study because none of the patient's family members had malignant tumors.

The treatment strategies for MPMTs depend on some factors, such as the tumor stage, patient's age and comorbidities. Furthermore, a multidisciplinary team management approach is necessary for personalized therapy. In metachronous MPMTs, the treatment always involves sequential therapy for each tumor, whereas in synchronous MPMTs, individualized and single treatment is decided upon after appropriate evaluation and consensus is reached by multidisciplinary team (Vogt et al., 2017). The patient underwent surgery and postoperative radiotherapy for synchronous esophageal and gastric carcinomas. Then, he underwent laparoscopic-assisted resection of right-sided colonic cancer and lysis of abdominal adhesions. By the time of submitting this manuscript, the patient had been well and no sign of recurrence or metastasis had been detected.

To the best of our knowledge, this case is the first one to clarify the genetic abnormalities of triple primary cancers of esophagus, stomach and colon in a Chinese patient. It may contribute to understanding the molecular pathogenesis of multiple primary digestive malignancies and providing valuable treatment strategies for the similar patients in the future. The limitation of this study is that NGS-based multi-gene panel couldn't identify all genetic predispositions like epigenetic modifications and other CNVs.

## Data Availability Statement

All of the data supporting the findings in this study are available upon reasonable request from the corresponding author (Yuedong Wang).

## Ethics Statement

The studies involving human participants were reviewed and approved by the Ethics Committee of the Second Affiliated Hospital Zhejiang University School of Medicine. Written informed consent for participation in the study, publication of clinical details and images was obtained from the patient.

## Author Contributions

YW: conceptualization. XZ: writing-original draft. SC and EM: conducting bioinformatics analysis. LH and KS: writing-review and editing. All authors contributed to the article and approved the submitted version.

## Conflict of Interest

SC and EM are employees at ChosenMed Technology. The remaining authors declare that the research was conducted in the absence of any commercial or financial relationships that could be construed as a potential conflict of interest.
